# Total flavonoids extracted from *Nervilia Fordii* function in polycystic ovary syndrome through IL-6 mediated JAK2/STAT3 signaling pathway

**DOI:** 10.1042/BSR20181380

**Published:** 2019-01-03

**Authors:** Yanyuan Zhou, Liang Lv, Qinghua Liu, Jiale Song

**Affiliations:** 1 College of Pharmacy, Guilin Medical University, Guilin, Guangxi, P.R. China; 2 College of Public Health, Guilin Medical University, Guilin, Guangxi, P.R. China

**Keywords:** flavonoids, interleukin-6, ovary, polycystic ovary syndrome

## Abstract

Large doses of flavonoids could cure many diseases with no serious side effects. However, the role of flavonoids in the treatment of polycystic ovary syndrome (PCOS) has not been reported. Therefore, total flavonoids extracted from *Nervilia Fordii* were selected to explore its therapeutic efficiency in PCOS. PCOS rat model was constructed to explore the role of total flavonoids in the treatment of PCOS. ELISA was used to assess the changes of ovulation function under the treatment of total flavonoids with or without exogenous interleukin-6 (IL-6). Western blot, real-time PCR and immunohistochemistry were carried out to assess the related molecular mechanisms. We explored that total flavonoids obviously increased the serum levels of follicle-stimulating hormone (FSH), and sharply decreased the serum levels of luteinizing hormone (LH), testosterone (T) and insulin (INS) in the PCOS-IR rats via partly inhibiting the activation of JAK2/STAT3 pathway, partially up-regulating the IL-6 expression and partially down-regulating the suppressor of cytokine signaling 3 (SOCS3) expression in ovaries of PCOS rats. The effect of total flavonoids on estrous cycles, serum levels of FSH, LH, T and INS were partially attenuated by IL-6 in PCOS rat model. Moreover, IL-6 significantly reversed the effect of total flavonoids on the phosphorylation of JAK2/STAT3, the expression of IL-6 and SOCS3 in ovaries of PCOS rats. Total flavonoids extracted from *Nervilia Fordii* might induce the expression of IL-6 in ovary and act as a potential therapeutic drug for the treatment of PCOS.

## Introduction

Polycystic ovary syndrome (PCOS) is a common endocrine metabolic syndrome in gynecological clinic, which is characterized by polycystic ovary, ovulation disorder and hyperandrogenemia [1,2]. It is generally believed that the incidence of PCOS is 5–10%, which is higher in adolescents and women of childbearing age [3,4]. In recent years, the incidence of polycystic ovary syndrome has been significantly increased. The clinical manifestations of polycystic ovary syndrome were menstrual disorders, infertility, thin sores, obesity, hairy and so on, it seriously affected the quality of life in women [5–7]. Therefore, how to effectively treating polycystic ovary syndrome became one of the hot spots in current medical research practice.

Flavonoids are important components of foods that are commonly consumed including vegetables, fruits and herbal ingredients. It is well known that flavonoids are bioactive polyphenols that are considered to be related with anti-inflammatory, anti-aging, antifungal and especially anticancer activities due to their highly reactive oxygen radicals including alkoxyl, hydroxyl or peroxide radicals as well as their effective suppression of lipid peroxidation in micelle systems [8–10]. Moreover, many clinical trials have been carried out with large doses of flavonoids to prevent disease, and these studies have found no serious side effects [11]. Literatures have reported that *Nervilia Fordii* has the efficacy of phlegm hemoptysis, detoxification and detoxication [12,13]. However, the role of flavonoids in the treatment of polycystic ovary syndrome has not been reported. Therefore, total flavonoids extracted from *Nervilia Fordii* were selected for subsequent experimental studies.

Here, we used the insulin and human chorionic gonadotropin to establish the PCOS rat model and the role of total flavonoids extracted from *Nervilia Fordii* in PCOS was explored. Then, the serum levels of luteinizing hormone (LH), testosterone (T) and insulin (INS) in the PCOS-IR rats were measured. The JAK2/STAT3 pathway and the expression of IL-6 and SOCS3 were detected, respectively. The present study aims to provide an effective and less side-effect method for the treatment of patients with PCOS.

## Materials and methods

### Animal models and treatments

The female SD rats (25-day-old) were randomly divided into the control group (*n*=8) and PCOS groups (*n*=56). The PCOS group was daily subcutaneous injected with DHEA (6 mg/100 g/d, 0.2 ml/mouse in sesame oil, Sigma) for 20 consecutive days while the vehicle control group was injected with 0.2 ml of sesame oil daily. After 20 days of continuous injection, the changes of vaginal exfoliative cytology in rats were observed by toluidine blue staining. The successfully construction of PCOS rat model was identified by vaginal epithelial cells exhibited keratosis for 10 consecutive days. After 12 hours of fasting, the orbital veins blood of the PCOS rats was obtained and the fasting blood glucose (FBG) and fasting insulin (FINS) were measured. According to the homeostasis model assessment of insulin resistance (HOMA-IR) method, HOMA-IR = FBG (mmol/l) × FINS (mU/l)/22.5, the PCOS rats with HOMA-IR > 2.8 were regarded as insulin resistance (PCOS-IR rat models) and used for the further experiments.

In the experiments, expect from the control group, the PCOS-IR rats were randomly divided into seven groups with eight rats in each group: (i) PCOS-IR group, (ii) PCOS-IR group treated with total flavoniods (50 mg/kg, low dose) extracted from *Nervilia Fordii*, (iii) PCOS-IR group treated with total flavoniods (100 mg/kg, middle dose), (iv) PCOS-IR group treated with total flavoniods (200 mg/kg, high dose), (v) PCOS-IR group treated with metformin (100 mg/kg), (vi) PCOS-IR group treated with IL-6, (vii) PCOS-IR group treated with IL-6 and total flavonoids (200 mg/kg, high dose). The drugs were administered by gavage for 28 consecutive days, meanwhile the control group and PCOS model group were given matching normal saline. In addition to gavage, the rats in IL-6 group were also subcutaneous injected with rat recombinant IL-6 protein (2 μl) for 28 consecutive days. After 28 days of continuous administration, the changes of the indicators of rats were analyzed in various groups.

This research was approved by the Experimental Animal Ethics Committee of the Guilin Medical University (Guangxi, China) with an approval No. 2017-0001.

### Toluidine blue stain

The changes of vaginal exfoliative cytology in rats were determined by toluidine blue stain. First, the aseptic cotton swab was soaked in the physiological saline then put it on the rat’s vaginal wall and smeared it clockwise. Second, taken out the cotton swab and smeared it on the slide (Servicebio, MA) in the same direction then the cells on the slide were fixed with 4% paraformaldehyde for 15 min. Last, the vaginal smear were stained by toluidine blue (Servicebio, MA) according to the manufacturer’s guideline. Images were captured using a microscope imaging system (Nikon, Japan).

### ELISA assay

The orbital veins blood of the rats was obtained after 20 days of subcutaneous injection of DHEA or 28 days of continuous administration. The sera separated from blood samples were stored at −80°C until detected. The FSH, LH, T and INS in sera were determined by ELISA according to the manufacturer’s guideline. The testing kits for FSH, LH, T and INS were purchased from Elabscience (Elabscience, U.S.A.).

### Western blot analysis

Solid tissues (40 mg) were shredded and lysed using RIPA buffer (Boston, U.S.A.) that was reconstituted with protease inhibitors (Roche Diagnostics, U.S.A.) and phosphatase inhibitors (Roche, U.S.A.), and then homogeneously broken tissues in a homogenizer. The BCA Protein Assay kit (Pierce, U.S.A.) was used to measure the protein concentration. The protein samples (20 μg/lane) were subjected to SDS-PAGE and transferred onto PVDF membranes (Millipore, U.S.A.). Incubated with antibodies against STAT3 (cell signal technology, 1:2000), p-STAT3 (CST, 1:1000), JAK2 (CST, 1:5000), p-JAK2 (CST, 1:1000), IL-6 (Santa, 1:1000), SOCS3 (CST, 1:1000) or GAPDH (Abcam, 1:10000). HRP-conjugated goat anti-rabbit secondary antibodies (BOSTER, 1:20000) or HRP-conjugated Goat anti-mouse secondary antibodies (BOSTER, 1:20000) were used and the band was visualized using an enhanced chemiluminescence technique (ECL, Amersham Biosciences, U.S.A.).

### Real-time PCR

The expression of IL-6 and SOCS3 were investigated via real-time PCR assay. Taken proper amount of tissues to lap in liquid nitrogen, then RNA was extracted by Trizol (Takara, China). The reverse transcription was performed using Bestar qPCR RT Kit (DBI Bestar, Germany). To quantify gene amplification, real-time PCR analysis was performed using an Agilent StratageneMx3000PSequence Detection System in the presence of Bestar® SybrGreen (DBI Bestar, Germany). The cycling parameters were 95°C for 2 min, followed by 40 cycles of 94°C for 20 s, 57°C for 20 s and 72°C for 20 s, with a final extension at 72°C for 10 min, a melting curve analysis was subsequently conducted. The relative expression levels (defined as fold changes) of the target genes were normalized to the folds of the corresponding control cells. The primer sequences are as follows: GAPDH forward primer: 5′-CCTCGTCTCATAGACAAGATGGT-3′, reverse primer: 5′-GGGTAGAGTCATACTGGAACATG-3′; IL-6 forward primer: 5′-AATAGTCCTTCCTACCCCAAC-3′, reverse primer: 5′-CGAGTAGACCTCATAGTGACCTT-3′; SOCS3 forward primer: 5′-TTTACCACCGACGGAACCTT-3′, reverse primer: 5′-CGACAAAGATGCTGGAGGGTA-3′.

### Immunohistochemical staining (IHC)

The expression of IL-6, SOCS3, p-STAT3 and Ki67 in ovarian tissues were determined by immunohistochemistry staining according to the manufacturer’s guideline. The ovary tissue sections were obtained using a slicing system (Leica, Newcastle, U.K.). Primary antibody for Ki67 was purchased from abcam (Cambridge, MA). The sections were first incubated with the primary antibodies overnight at 4°C and then HRP-conjugated secondary antibody at room temperature. After a final wash, the slides were incubated with diaminobenzidine substrate (Beyotime, China) by using the avidin-biotin HRP system. Images were captured using a microscope imaging system (Nikon, Japan). IHC score was determined according to the percentage of positively staining cells (negative staining, IHC score 0; <20% of positively staining cells, IHC score 1; 20–50% of positively staining cells, IHC score 2; >50% of positively staining cells, IHC score 3). IHC evaluation was performed by two pathologists who were blind to the clinical and pathological characteristics associated with the samples.

### Statistical analysis

All experiments were repeated at least three times. The data are presented as the mean ± SEM. Student’s *t*-test and one-way ANOVA with the Newman–Keuls post test were applied. Statistical analysis was carried out by GraphPad Prism Version 3.0 (San Diego, CA, U.S.A.). Differences were considered significant at *P*<0.05.

## Results

### Irregular estrous cycle and insulin resistance in PCOS rats model

**Figure 1:**
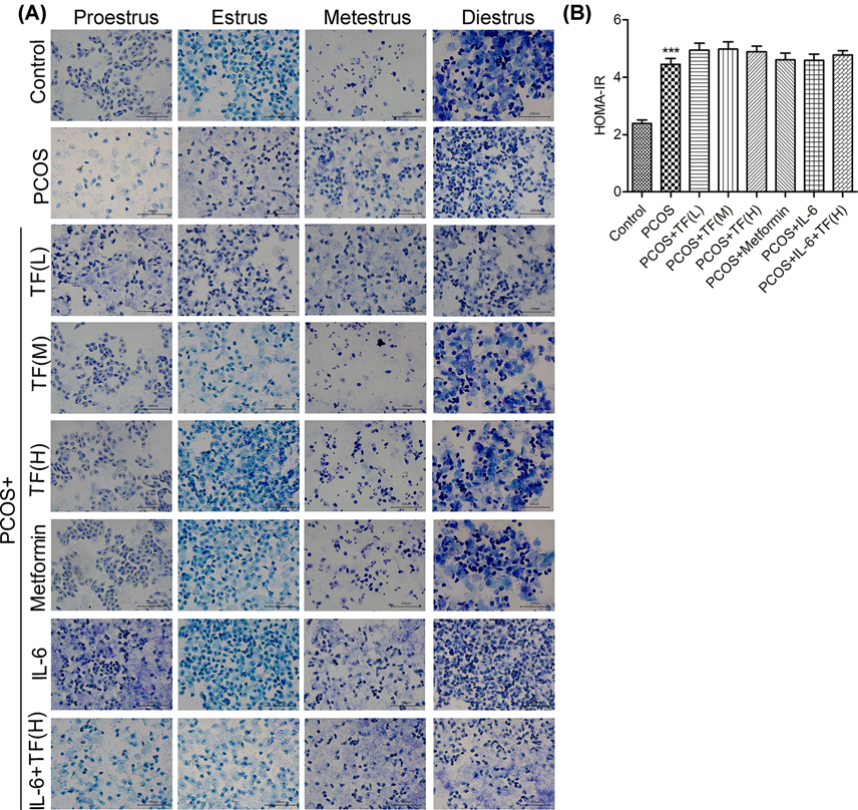
Irregular estrous cycle and insulin resistance in the PCOS rat model To explore the effect of total flavonoids on estrous cycle, sex hormone levels and insulin levels in PCOS-IR rats model, the rats were randomly divided into eight groups: Control group, PCOS-IR group [PCOS], PCOS-IR group treated with total flavoniods (50 mg/kg, low dose) extracted from nervilia fordii [PCOS+TF(L)], PCOS-IR group treated with total flavoniods (100 mg/kg, middle dose) [PCOS+TF(M)], PCOS-IR group treated with total flavoniods (200 mg/kg, high dose) [PCOS+TF(H)], PCOS-IR group treated with metformin (100 mg/kg) [PCOS+Metformin], PCOS-IR group treated with IL-6 [PCOS+ IL-6] and PCOS-IR group treated with IL-6 and total flavoniods (200 mg/kg, high dose) [PCOS+ IL-6+ TF(H)]. (**A**) The changes of vaginal exfoliative cytology in rats were determined by toluidine blue stain. (**B**) The orbital veins blood of the PCOS rats was obtained and measured homeostasis model assessment of insulin resistance (HOMA-IR) by detection of the fasting blood glucose and fasting insulin; ***P<0.001 vs. Control.

The construction of PCOS model was evaluated from two aspects, sexual cycle change and insulin resistance (IR). In control group, proestrus smear was mainly composed of nuclearted epithelial cells, estrussmear was mainly composed of cornified cells, metestrus smear was composed of same ratio of leukocytes, epithelial cells and cornified cells, diestrussmear was majorly composed of leukocytes (Figure 1A). The results showed that the rats in control group had a normal estrous cycle. However, the vaginal smear in PCOS model group was mainly composed of cornified cells in all phases of estrous cycle. These data also showed the vaginal exfoliative cells in PCOS model exhibited vaginal keratosis and an irregular estrous cycle (Figure 1A). All the results demonstrated that the rat PCOS model was constructed successfully.

After 12 h of fasting, we measured the FBG and FINS (Figure 1B). The insulin resistance was calculated according to HOMA-IR method [14]. The data showed that the value of HOMA-IR was approximately equal to 2.4 in control group while the HOMA-IR was approximately 4.5 in PCOS model group (Figure 1B). The PCOS rats exhibited HOMA-IR > 2.8 were regarded as insulin resistance. All the results showed that the PCOS model with insulin resistance in SD rats (PCOS-IR rat model) was constructed successfully. The PCOS-IR rat model was used for the further experiments.

### Effect of total flavonoids treatment on estrouscycles, serum levels of FSH, LH, testosterone and insulin in PCOS-IR rat model

To evaluate the effect of total flavonoids on estrous cycle in the PCOS-IR rats, the drugs were administered by gavage for 28 consecutive days while the control and PCOS-IR groups were given matching normal saline. As shown in Figure 1, compared with control group, low-dose total flavonoids had no effect on the estrous cycle, middle-dose total flavonoids had slight effect on the estrous cycle, while high dose total flavonoids as well as metformin could recover estrous cycle in PCOS-IR rats with insulin resistance. The results showed that total flavonoids could recover estrous cycle in PCOS-IR rats in a dose-dependent manner, indicating that the estrous cycle was associated with glucose metabolism disorders in PCOS-IR rats.

**Figure 2:**
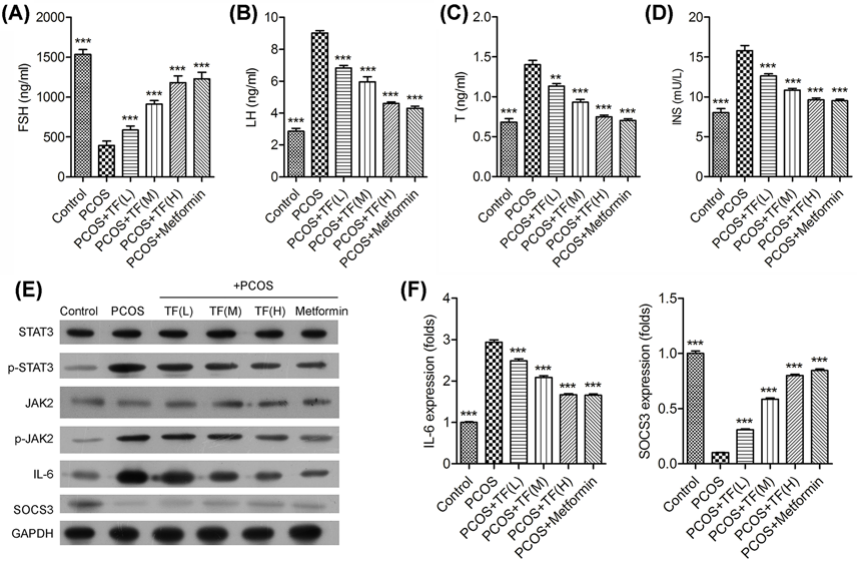
Effect of total flavonoids treatment in PCOS-IR rat model The orbital veins blood of the rats was obtained, the serum levels of FSH (**A**), LH (**B**), testosterone (**C**) and insulin (**D**) in sera were determined by ELISA, respectively. (**E**) The expression of JAK2, STAT3, IL-6 and SOCS3 in ovaries of PCOS rats were analyzed by Western blot. (**F**) The expression of IL-6 and SOCS3 were analyzed by real-time PCR; **P<0.01, ***P<0.001 vs. PCOS.

The serum levels of FSH, LH, T and INS levels in each group were shown in the Figure 2. Compared with control group, there was a significant decrease in serum levels of FSH in PCOS-IR rats (Figure 2A), and the significant increase in serum levels of the LH, T and INS in PCOS-IR rats (Figure 2B–D). Moreover, total flavonoids significantly increased the serum levels of FSH in PCOS-IR rats in a dose-dependent manner, meanwhile sharply decreased the serum levels of LH, T and INS in PCOS-IR rats. Among the drug-treated groups, both high dosage of total flavonoids and metformin obviously increased the serum levels of FSH, and high dosage of total flavonoids and metformin mostly decreased the serum levels of LH, T and INS levels in PCOS-IR rats.

### Total flavonoids inhibited the phosphorylation of JAK2/STAT3 pathways and regulated the expression of IL-6 and SOCS3 in ovaries of PCOS rats

To further study the related molecular mechanism of total flavonoids in the treatment of PCOS rats, we examined the expression and phosphorylation of JAK2, STAT3 and the expression of IL-6 and suppressors cytokine signaling 3 (SOCS3) in the ovaries of PCOS rats. As shown in Figure 2, there was no significant difference in the expression of JAK2 and STAT3 among all the groups, while the phosphorylation of JAK2 and STAT3 significantly increased in the PCOS rat model group when compared with the control group (Figure 2E). Moreover, the phosphorylation of JAK2 and STAT3 was significantly decreased in the high dosage of total flavonoids and metformin-treated PCOS rats. In addition, the expression of IL-6 and SOCS3 in ovaries of PCOS rats was also determined by Western blotting and real-time PCR methods. Results showed that the expression of IL-6 was significantly increased in ovaries of PCOS rat model group when compared with control group, while the expression of IL-6 was significantly decreased in the total flavonoids high dosage and metformin-treated PCOS rats (Figure 2F). Meanwhile, the expression of SOCS3 was contrary to the results of IL-6, the expression of SOCS3 was significantly increased in PCOS rats treated with high dosage of total flavonoids and metformin. This effect was coincided with the decrease in IL-6 expression and increase in SOCS3 expression.

### The effect of total flavonoids on the expression of IL-6, SOCS3, Ki67 and phosphorylation of STAT3 in ovarian tissues of PCOS rats

**Figure 3:**
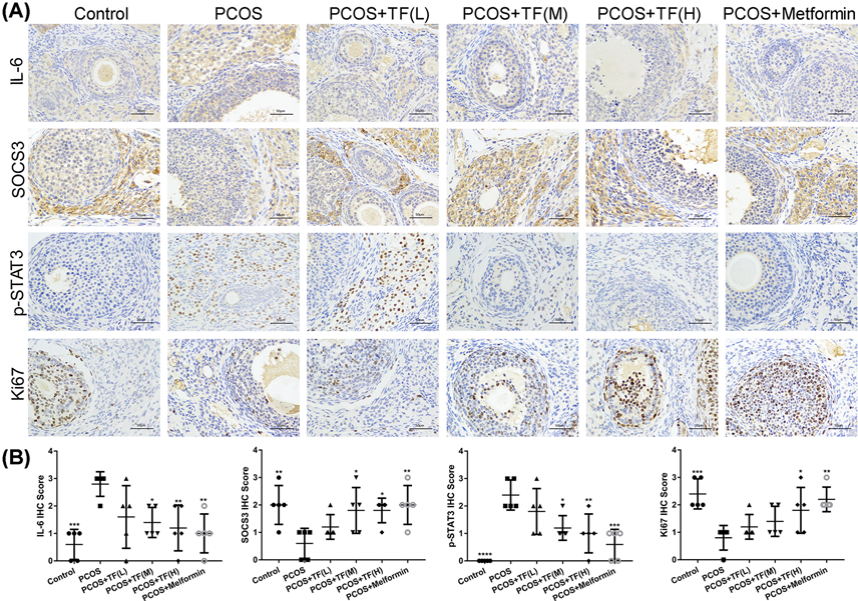
The effect of total flavonoids on the expression of IL-6, SOCS3, Ki67 and phosphorylation of STAT3 in ovarian tissues of PCOS rats (**A**) The expression of IL-6, SOCS3, Ki67 and phosphorylation of STAT3 in ovaries of PCOS rats were detected by IHC. (**B**) The changes of IL-6, SOCS3, p-STAT3 and Ki67 expression in ovaries of PCOS rats after treated with different concentrations of total flavonoids and Metformin. IHC score was determined according to the percentage of positively staining cells; *P<0.05, **P<0.01, ***P<0.001, **** *P*<0.0001 vs. PCOS.

As shown in Figure 3, IHC was performed on the ovarian tissue slides to observe the levels of IL-6, SOCS3, Ki67 and phosphorylation of STAT3 among all the groups, thereby to determining the therapeutic effect of total flavonoids on inflammatory cytokines and cell proliferation of the PCOS rats. As shown in Figure 3, the IL-6 and phosphorylation of STAT3 immunostaining was markedly increased in the PCOS rat model group when compared with that in control group. However, IL-6 and phosphorylation of STAT3 immunostaining were significantly decreased in the total flavonoids high dosage and metformin-treated PCOS rats (Figure 3A,B). In contrast, the SOCS3 and Ki67 immunostaining in the PCOS rat model group were lower than that in control group, and the expression of SOCS3 and Ki67 were significantly increased in the total flavonoids high dosage and metformin-treated PCOS rats (Figure 3A,B).

### The effect of total flavonoids on estrous cycles, serum levels of FSH, LH, testosterone and insulin were attenuated by IL-6 in PCOS-IR rats model

To explore the effect of IL-6 on sexual cycle estrous cycles of PCOS model, the rats were treated with IL-6, or treated with IL-6 and total flavoniods (200 mg/kg, high dose). The drugs were administered by gavage feeding for 28 consecutive days while the control group and PCOS model group were given matching normal saline. In addition to gavage feeding, the rats in IL-6 group were also subcutaneous injected with rat recombinant IL-6 protein (2 μl) for 28 consecutive days. The vaginal smears demonstrated that the estrous cycles were irregular in PCOS rat model group when compared with control group, and total flavonoids high dosage treated PCOS rats recovered the estrous cycles in PCOS rats (Figure 1A). In addition, the ability of total flavonoids to recover the estrous cycle was eliminated by IL-6 in PCOS rat group. All the results indicated that the effect of total flavonoids on estrous cycle could be mediated via inhibiting IL-6 expression.

**Figure 4:**
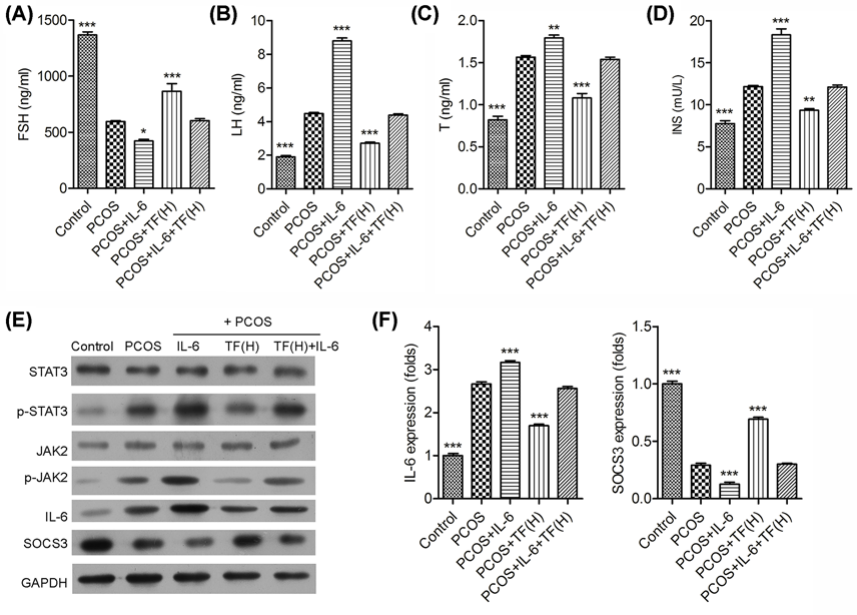
Effect of total flavonoids were attenuated by IL-6 in PCOS-IR rats model The orbital veins blood of the rats was obtained, the serum levels of FSH (**A**), LH (**B**), testosterone (**C**) and insulin (**D**) in sera were determined by ELISA, respectively. (**E**) The expression of JAK2, STAT3, IL-6 and SOCS3 in ovaries of PCOS rats were analyzed by Western blot. GAPDH was used as an internal control. (**F**) The expression of IL-6 and SOCS3 were analyzed by real-time PCR; *P<0.05, **P<0.01, ***P<0.001 vs. PCOS.

We further found that IL-6 could significant decrease in serum levels of the FSH (Figure 4A), and significant increase in serum levels of the LH (Figure 4B), T (Figure 4C) and INS (Figure 4D) in PCOS-IR rat models. Moreover, the effect of total flavonoids on increasing the serum levels of FSH and decreasing the serum levels of LH, T and INS were attenuated by IL-6 in PCOS-IR rat model. All the results demonstrated that the therapeutic effect of total flavonoids on serum levels of the FSH, LH, T and INS were attenuated by IL-6 in PCOS-IR rat model.

### Effect of total flavonoids on the expression of JAK2/STAT3, IL-6 and SOCS3 were reduced by IL-6 in ovaries of PCOS rats

As shown in Figure 4, there was no significant difference in the expression of JAK2 and STAT3 among all the groups, but the phosphorylation of JAK2 and STAT3 were significantly increased in the IL-6 treated PCOS rats when compared with the PCOS rats (Figure 4E). Moreover, PCOS rats treated with IL-6 and total flavonoids had a higher level of JAK2 and STAT3 phosphorylation than that only treated with total flavonoids. In addition, the expression of IL-6 was significantly increased in IL-6 treated PCOS rats when compared with PCOS rats; meanwhile, the expression of IL-6 showed a higher increase in PCOS rats treated with IL-6 and total flavonoids than that only treated with total flavonoids (Figure 4E,F). What’s more, the expression of SOCS3 was contrary to the results of IL-6, the expression of SOCS3 was significantly decreased in PCOS rats treated with IL-6 and total flavonoids when compared with that only treated with total flavonoids.

### The effect of total flavonoids on the expression of IL-6, SOCS3, Ki67 and phosphorylation of STAT3 in ovarian tissues of PCOS rats were partially regulated by IL-6

**Figure 5:**
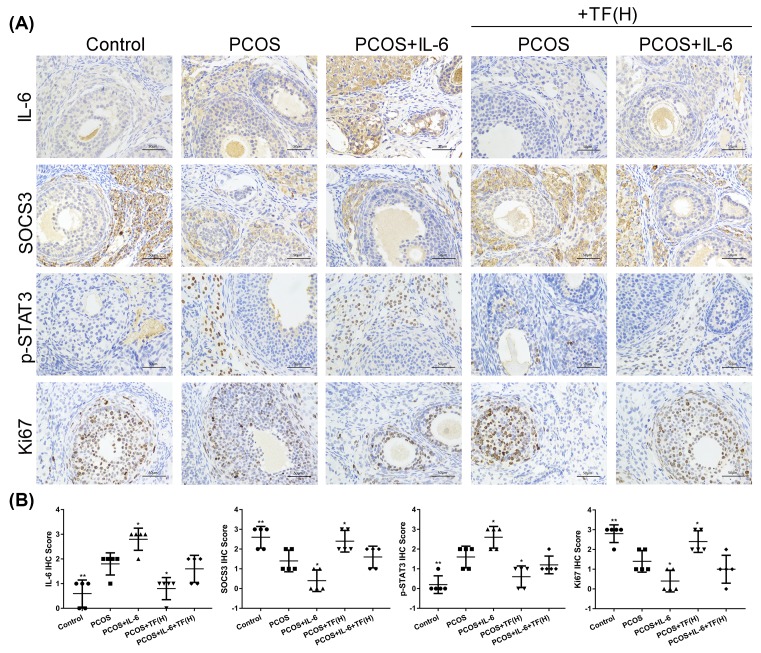
The effect of total flavonoids on the expression of IL-6, SOCS3, Ki67 and phosphorylation of STAT3 in ovarian tissues of PCOS rats were regulated by IL-6 (**A**) The expression of IL-6, SOCS3, Ki67 and phosphorylation of STAT3 in ovaries of PCOS rats were detected by IHC. (**B**) IHC score was determined according to the percentage of positively staining cells. The changes of IL-6, SOCS3, p-STAT3 and Ki67 expression in ovaries of PCOS rats after treated with IL-6 combined with or without high dose of total flavonoids; *P<0.05, **P<0.01 vs. PCOS.

As shown in Figure 5, the levels of IL-6, SOCS3, Ki67 and phosphorylation of STAT3 were detected by IHC assay on the ovarian tissues slides among all groups to determine the effect of IL-6 on PCOS rats treated with total flavonoids. The IL-6 and phosphorylation of STAT3 immunostaining in the IL-6 treated PCOS rats were markedly higher than that in PCOS rats (Figure 5). In addition, the IL-6 and phosphorylation of STAT3 immunostaining were significantly increased in PCOS rats treated with IL-6 combination with total flavonoids when compared with the PCOS rats only treated with total flavonoids. By contrast, the SOCS3 and Ki67 immunostaining was much lower in IL-6 associated with total flavonoids treated PCOS rats than in the total flavonoids treated PCOS rats. All data showed that the influence of total flavonoids on the expression of JAK2/STAT3, IL-6 and SOCS3 was partially regulated by IL-6 in ovaries of PCOS rats.

## Discussion

PCOS is an extremely complex endocrine and metabolic disease. Although the understanding of the disease has been achieved in many aspects, its etiology has not been fully elucidated, and the treatment has not achieved breakthrough progress. At present, clinical treatment of PCOS is mainly combined with drugs and surgery. Although the drug treatment of PCOS could improve the clinical symptoms, there are many deficiencies, such as inducing the endometrium thinning, increasing the risk of thrombosis and damaging the liver function of patients with hepatitis B [15–17]. Surgical treatments mainly include laparoscopic ovary perforation and ovary wedge resection [18,19]. The biggest side effects of surgical treatment are the destruction of the ovary and the depletion of the reserve follicles, which lead to the premature ovarian failure in many patients [18,20]. Therefore, the surgical treatment of PCOS is rarely used by clinicians. In recent years, more and more researchers have turned their attention to traditional Chinese medicine (TCM). Clinical studies have shown that TCM treatment of this disease has the advantages of natural non-toxic, long-lasting, comprehensive efficacy, patient compliance, and become more and more popular by people [21–23].

Flavonoids, the main ingredient in *Nervilia Fordii*, have good effects on anti-inflammatory, analgesic, immunity enhancing, heat relieving, cough relieving etc [24,25]. In our study, we found that total flavonoids extracted from *Nervilia Fordii* significantly increased the serum levels of FSH and sharply decreased the serum levels of LH, T and INS levels in PCOS-IR rats, meanwhile high dose total flavonoids could recover estrous cycle in PCOS-IR rats with insulin resistance, indicating the total flavonoids could restore the ovulation function.

In recent years, PCOS has been found to be a chronic low-grade inflammation, which is closely related to long-term complications such as diabetes and cardiovascular disease [26]. IL-6, as a common chronic inflammatory factor, plays an important role in inflammatory response, immune regulation and hematopoietic regulation. Although IL-6 does not directly promote the production of testosterone secreted by follicular cell, it can increase the expression of androgen receptor in ovarian tissue, thus indirectly increasing the activity of androgen and becoming one of the mechanisms of PCOS occurrence [27]. Studies have confirmed that IL-6 was closely related to type 2 diabetes and insulin resistance. In obese and type 2 diabetics patients, up-regulation of IL-6 levels were associated with elevated blood sugar levels, low sugar tolerance and decreased sensitivity to insulin [28,29]. Here, we demonstrated that the estrous cycle of PCOS rats was disordered, and IL-6 expression was significantly increased in ovarian tissues of PCOS rats, total flavonoids could recover estrous cycle and decrease the level of IL-6 that increased in PCOS rats, indicating that total flavonoids might treat PCOS through down-regulating the IL-6 expression and promoting the recovery of ovarian function.

Studies have found that the expression level of serum leptin in PCOS was significantly higher than that of healthy women with the same physical quality and normal ovulatory cycle. In the process of intracellular signal transduction of leptin, the JAK/STAT3 signaling pathway was the main regulation pathway [30]. Moreover, Maliqueo et al. also proved that the placental STAT3 signaling was activated in women with PCOS [31]. In our study, we also demonstrated that the phosphorylation levels of JAK2 and STAT3 were remarkably increased in PCOS rats, and this effect was enhanced by IL-6 treatment and partially rescued by the treatment of total flavonoids. Moreover, we also verified that SOCS3 (suppressor of cytokine signaling 3), one of SOCS3 proteins that were considered to be the main negative regulators of the JAK/STAT signal pathway, was sharply suppressed in PCOS rats, but partially reversed under the treatment of total flavonoids and further demonstrated that total flavonoids played its therapeutic efficiency via regulating the JAK2/STAT3 pathway.

Here, our data provided that total flavonoids extracted from *Nervilia Fordii* could recover the estrous cycle, decrease the serum levels of FSH, increase the serum levels of LH, T and INS through partly inhibiting the activation of JAK2/STAT3 pathways and partially regulating the IL-6 and SOCS3 expression in ovaries of PCOS rats. All these results provided that total flavonoids extracted from *Nervilia Fordii* might have the therapeutic efficiency in the treatment of PCOS.

## Abbreviations

FBG: fasting blood glucose
FINS: fasting insulin
FSH: follicle-stimulating hormone
IL-6: interleukin-6
LH: luteinizing hormone
PCOS: polycystic ovary syndrome
SOCS3: suppressor of cytokine signaling 3
